# Jammin’ the Blues: Experiencing the “Good Life”

**DOI:** 10.3389/fpsyg.2021.648007

**Published:** 2021-06-07

**Authors:** Ruth A. Debrot

**Affiliations:** Department of Music Education, Boston University, Boston, MA, United States

**Keywords:** eudaimonia, music education, older adults, popular music, well-being

## Abstract

The purpose of this study was to examine the characteristics, attitudes, and perceptions of older musicians who participated regularly in a local blues jam. Six core dimensions of eudaimonic well-being and their conceptual foundations provided a framework for examining the way that music-making contributes to subjective well-being during the lifespan of an individual. The following research questions guided this investigation: (1) In what ways do biographical factors and engagement with music influence the lives of older adult blues/rock musicians who participate in a local blues jam? (2) What implications for subjective well-being with regard to music learning might be used to inform music education practices? Interviews and observations over a 2-month period provided data for understanding how lived experiences impacted personal well-being, and musical growth and development over time. Findings suggested that eudaimonic well-being is the result of active engagement in human activities that are goal-directed and purposeful, and a good life involves the self-realization of individual dispositions and talents over a lifetime. Implications for music education include individualized pedagogical approaches that encourage learners to discover a sense of well-being in and through music.

## Introduction

The blues jam at The Tavern on the Hill is open to musicians of all ages, but most participants are baby boomers. The jam provides an opportunity to gather, socialize, have a few drinks, and participate in informal, improvisational music-making. Bringing an instrument or set of drumsticks to the jam indicates a willingness to perform. Hot Rod Billie, the owner of The Tavern, motions people carrying instruments toward a clip board so they can sign-up and be called to the stage. Participation is encouraged and expected.

Rooted in jazz and funk, jamming is an interactive and improvisational musical practice. Turino (2009) described jamming as a groove-based practice centered on ethical values of inclusivity that are derived from “enticing people to join in” (p. 110). Jamming is a form of musical communication involving dialectic relationships that are predicated on musical flow and resistance, and response to self and other (Michaelson, 2013; Brink, 2018). From a cognitive standpoint, jamming requires embodied forms of intersubjective understanding, conceptual knowledge related to Piaget’s (1952) notion of *schema*, cognitive and executive functioning, and the ability to carry out systematic and prioritized attention (Doffman, 2011). Musicians participate in jam sessions to interact with others, further their musical skills, make themselves known in the community, and to feel good.

This study grew out of my desire to understand how participatory music-making, in the context of a local blues jam, fostered a subjective sense of personal meaning, purpose, and fulfillment in the lives of older musicians. Within the realm of popular music, Frith (1996) suggested, “Music isn’t a way of expressing ideas; it’s a way of living them” (p. 111). Turino (2008) discovered that cultural participation in expressive artistic experiences and music performances can lead to a sense of a “more deeply lived life” (p. 18). I engaged in this study to examine how peoples’ lived experiences influenced their music participation, music learning, personal growth, and well-being over a lifetime.

## Framework

Contemporary scholars have proposed that active musical engagement leads to well-being and personal fulfillment, a philosophical concept known as *eudaimonia* (Elliott and Silverman, 2014; Boyce-Tillman, 2020; Smith and Silverman, 2020). Rooted in the philosophy of Aristotle, eudaimonia is:

*A multidimensional term that means full human flourishing: a “good life” of significant, enjoyable, and meaningful work and leisure; personal and community health and well-being; virtue; and fellowship, self-worth, and happiness for the benefit of oneself and others. (Elliott and Silverman, 2014, p. 59)*

It is important to note that when he wrote *Nichomachean Ethics* (Aristotle, 1980) more than 2000 years ago, Aristotle was not so much concerned with “well-being,” but more “human flourishing.” In doing so, he established principles for how to live a good life (Ryff and Singer, 2008). Notably, principles for how to live a good life, according to Aristotelian ethics, did not refer to hedonistic forms happiness or amusement. Rather, Aristotle spoke of “activity of the soul in accordance with virtue” (Aristotle, 1980, p. 11) Contemporary philosophers have maintained that “human flourishing and self-reflective happiness are the rewards of a life of virtue” (Silverman and Elliott, 2016; Smith and Silverman, 2020, p. 2). Eudaimonic well-being is the result of active engagement in human activities that are goal-directed and purposeful, and a good life involves the self-realization of individual dispositions and talents over a lifetime (Ryff and Singer, 2008; Silverman and Elliott, 2016).

The conceptual underpinnings that support psychological dimensions of eudaimonic well-being are important for understanding the developmental actions and challenges that individuals confront at various stages in life. Waterman et al. (2008) suggested that: “Eudaimonia, as a subjective state, refers to feelings present when one is moving toward self-realization in terms of the developing ones’ unique potentials and furthering ones’ purpose in living” (p. 42). Waterman (1993) used the terms self-realization and personal expressiveness as central, defining features, contending that eudaimonia is “activity expressing virtue” (p. 679).

**FIGURE 1:**
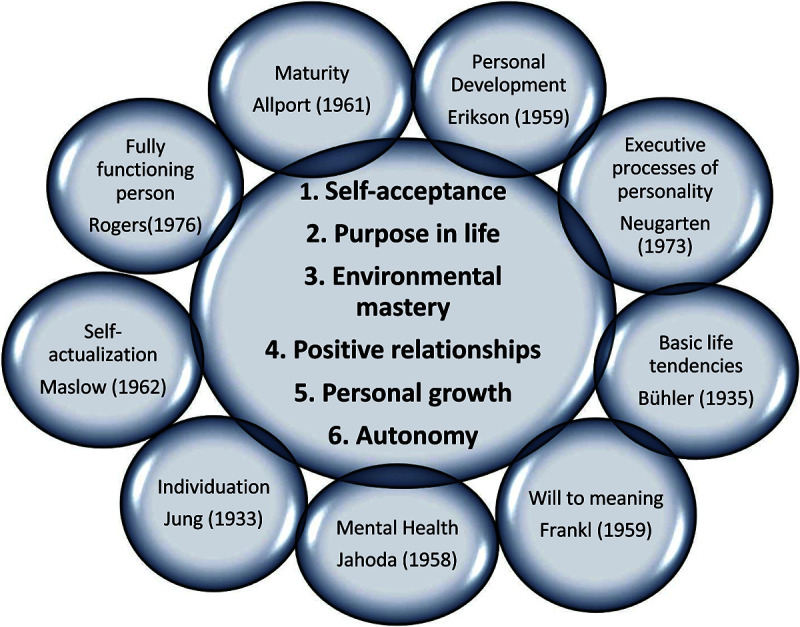
Six core dimensions of eudaimonic well-being and their conceptual foundations. Adapted from Ryff and Singer (2008, p. 20). Copyright by Springer 2016. Used with permission.

I employed Ryff and Singer’s (2008) six dimensions of well-being as a conceptual framework for examining ways that music-making might contribute to subjective well-being during the lifespan (see Figure 1). The following constructs of Vleioras and Bosma (2005) provided a framework for analysis:

• Self-acceptance; holding a positive opinion about oneself.
• Environmental mastery; being able to choose or create contexts appropriate forone’s psychological condition.
• Positive relations with others; having warm and trusting relationships and beingable to love.
• Purpose in life; having goals, intentions, and a sense of direction.
• Personal growth; continuous development of one’s potential.
• Autonomy; being self-determined and independent (p. 399).

The intersection of these constructs made it possible for me to examine individual well-being from the perspective of participants’ lived experiences with regard to individual, cultural and social engagement with music during the course of a lifetime.

### The Power of Music

Music is an integral part of human life. According to Levitin (2006), music is not simply a distraction or a pastime, but central to our identity and evolution as a species. Levitin (2006) revealed how music influences cognition by calling attention to powerful ways music is harnessed in advertising, films, ceremonies, and everyday human interactions. Humans create music and art in order to represent ideas that are important in life. Music plays an important and ongoing role in shaping people’s cognitive, social, and cultural understandings, which in turn, influences how people navigate their lives.

Although the wider benefits of music for younger people are well documented (Hallam, 2010), “less attention has been paid to the power of music making and learning in the lives of older adults” (Creech et al., 2013, p. 88). There is growing evidence corroborating the psychological, physical, and social benefits of music participation among older adults (Ernst and Emmons, 1992; Coffman and Adamek, 2001; Minichiello and Hayes, 2005; Joseph and Human, 2020). Music-making activities have been found to help people live longer and happier lives (Dabback and Smith, 2018). Listening to music and active music-making offer a vehicle by which older adults can express themselves and connect with others (Hallam et al., 2012). For older adults, active engagement with music engenders a sense of community, belonging, and social identity (Creech et al., 2013).

Hallam and Creech (2016) found that collaborative community music-making and -learning activities support cognitive, emotional, and mental health in adult settings. Both singing and playing instruments fostered a range of positive feelings among older adults (Lamont et al., 2018). Minichiello and Hayes (2005) suggested that “music provided individualized ways of knowing, understanding emotions, self, others, and expressing spirituality” (p. 449). Participants in choral singing groups reported an increased sense of community, personal achievement, and emotional well-being, coinciding with a decrease in loneliness, anxiety and depression (Dabback and Smith, 2018; Daykin et al., 2018; Joseph and Human, 2020). For over 30 years, an international organization called the New Horizons Bands has provided instrumental music-making opportunity for older, adult beginners (Ernst and Emmons, 1992; Coffman, 2002). Members of New Horizons claimed that “playing music lift[ed] their spirits” and that “being a valued member of a group and looking forward to concerts and other special events contribute[d] to positive attitudes and a sense of well-being” (Ernst and Emmons, 1992, p. 33). Findings from these studies confirmed the health benefits of formal music-making among older adults in group settings.

A number of music-related effects, drawing from the positive psychology movement, have been associated with eudaimonic well-being (Boyce-Tillman, 2020; Smith and Silverman, 2020). They include flow (Csikszentmihalyi, 1990; Lamont, 2011; Groarke and Hogan, 2016), peak experience (Maslow, 1971; Gabrielsson and Lindström, 2010), self-actualization (Maslow, 1962; Heintzelman, 2018), and transcendence (Minichiello and Hayes, 2005; Groarke and Hogan, 2016). The benefits of music making and learning are integral to well-being, virtue, and the importance of what it means to live a good life (Ryan et al., 2006; Elliott and Silverman, 2014). This study expands upon previous understandings regarding the positive benefits of music-making among older adults in informal, participatory, community-music settings.

### Needs and Behavior

Innate psychological needs drive human behavior (Murray, 1938; Hull, 1948; Deci and Ryan, 2000). Deci and Ryan (2000) investigated goal pursuits, human needs, and behavior using Self-Determination Theory (SDT) as a framework. The researchers contended that the need for competence, relatedness, and autonomy motivates humans to engage in goal pursuits and that the satisfaction of these pursuits facilitated psychological growth, integrity and well-being. Deci and Ryan differentiated between *performance* goals, which challenge one’s ability, and *learning* goals, which are about expanding one’s competencies. Goal-directed behaviors foster a sense of mastery, a construct associated with eudaimonic well-being.

### Lifelong Learning

Individual musical development is shaped by a range of cognitive, social and cultural factors that remain fluid and are constantly changing. Over the lifespan, musical development is characterized by “time-related changes in musical abilities, motivations, functions and musical activities” (Gembris, 2012, p. 367). Older non-professional musicians have unused musical potential, which can be activated at any time through learning and practice (Gembris, 2009). While information processing speed, memory or problem-solving begin to decrease during the third decade of life, these changes are typically offset by knowledge, experience, and social skills, which tend to increase during later years.

Lifelong learning is an ongoing process of “transforming experience into knowledge, skills, attitudes, values, emotions, beliefs and the senses” (Jarvis, 2002, p. 60). Smilde (2008) contended that lifelong learning shaped by four principles. The first is *autobiographical awareness* (Antikainen et al., 1996), which is related to a person’s sense of their musical identity. The second, *significant learning* (Rogers, 1969), refers to pivotal experiential moments during the life-course, that strengthen both individual and musical identity. *Critical incidents* (Giddens, 1991), refers to challenging, transformative moments, which influence one’s self-identity. The fourth determinant, attributed to Wenger (1998), denotes learning as *social participation*, where participants construct their personal and musical identities in relation to *communities of practice*. Smilde (2008) concluded that principles of lifelong learning enable musicians of any age to develop individualized, self-directed approaches to learning that correspond with their identities, foster self-exploration, and inspire self-reflection. The literature on lifelong learning supported the notion that virtually every older adult has the potential to develop new musical abilities and to expand on their existing skills and knowledge.

### Participatory Music-Making

Music-making in the context of a blues jam is focused on musical and social interaction. A distinguishing characteristic of participatory music-making is that there are no audience-artist distinctions. The success of a particular performance is judged on the level of participation achieved rather than the quality of the musical outcomes (Turino, 2008). When people attend the jam, it is assumed that everyone present can and should participate in the performance.

The inclusion of people with a wide variety of skills and interests during performances is important for encouraging participation (Turino, 2008). The differentiated musical tastes and abilities of the participants cultivates a unique dynamic, and some constraints, during performances. By the same token, musical interaction among novices and more skilled players provides scaffolding and ongoing musical challenges. In a participatory framework, having an ever-expanding set of challenges is critical so as to avert boredom. Price (2013) used the term *andragogy* to denote self-directed learning, which takes place during dialogic, participatory forms of music-making. When musical experiences are appropriately challenging and pleasurable, people return to the jam. As people learn new songs and engage with different people, they develop new skills.

The musical practices at a blues jam are predicated on the perspectives of individuals who come together and share their experience, skills, and perspectives. Camlin (2015) proposed that “the practices that emerge when human beings are in relation with each other, supporting each other’s development as human beings on a journey toward self-actualization, are by their very nature situated and local, and often very personal ways of making music” (p. 238).

The literature on participatory music-making supports the premise of this study, which is that “creative, collaborative processes, can enable any person, young or old, to build up a strong sense of who they are by empowering them to believe in themselves and take responsibility for their own lives and for those of others” (Renshaw, 2013, p. 4).

## Purpose

The purpose of this ethnographic study (Creswell, 2007) was to examine the characteristics and perceptions of a group of seven older musicians who participated regularly in a local blues jam. The research questions guiding the inquiry were:

1. In what ways do biographical factors and engagement with music influence the lives of older adult blues/rock musicians who participate in a local blues jam?
2. What implications for subjective well-being with regard to music learning might be used to inform music education practices?

I employed a biographical and social constructionist perspective (Rosenwald and Ochberg, 1992; Lamont, 2011) to understand how lived experiences impacted the personal well-being and musical growth and development of the participants over a lifetime. In order to accomplish this, I spent approximately 32 h in the field, documenting the interactions among the participants during the jam. I made video recordings of musical performances. I conducted 30–45-min, individual, semi-structured interviews with each participant. During interviews, I encouraged each person to discuss how they became involved with music, how they learned music, about their musical influences, and how they perceived the role music in their lives.

## Method

This study took place at a tavern in the Southeast region of Massachusetts. Data were collected over a 2-month period during December 2019 and January 2020. The Tavern on the Hill hosted an open-mic blues jam each Sunday evening from four to eight p.m. Musicians of all ages and abilities were encouraged to sign up to play alone or in friendship groups. The signup sheet, consisting of a legal pad on a clipboard, contained columns for each person to indicate their name, instrument, and songs they would like to play. The jam was facilitated by members of a house band, comprised of a guitar player, bassist, and drummer. All musicians were asked to play up to three songs per evening. The jam attracted regular participants, most of whom were over the age of 40.

### Data Collection and Analysis

A total of seven musicians, six males and one female, consented to participate in the study. I observed, interviewed, and made video recordings of two drummers, two guitarists, one bass player, one keyboard and saxophone player, and one vocalist. Per IRB protocols at Boston University, each participant granted me permission to interview them, use their real names, and to make video recordings of their performances. Interviews were recorded and transcribed. Member checks were completed.

Semi-structured, open-ended interviews took place in a private dining area at the back of the club during the jam session. Conversations were centered on the lived experiences of each participant and the role that music played during different stages of their lives. Recursive questioning (Minichiello et al., 1995) helped me encourage the participants to elaborate on topics that were related to the study, and to allow the participants to reflect openly regarding topics they thought might be relevant to the research.

Data analysis was an ongoing iterative and reflexive process (Stake, 1995). To gain additional insights, I expanded the number of participants from four to seven and engaged in a process known as *progressive focusing* (Parlett and Hamilton, 1972). Stake (1981) described progressive focusing as follows:

*Progressive focusing requires that the researcher be well acquainted with the complexities of the problem before going to the field, but not too committed to a study plan. It is accomplished in multiple stages: First observation of the site, then further inquiry, beginning to focus on the relevant issues, and then seeking to explain. (p.1)*

**FIGURE 2:**
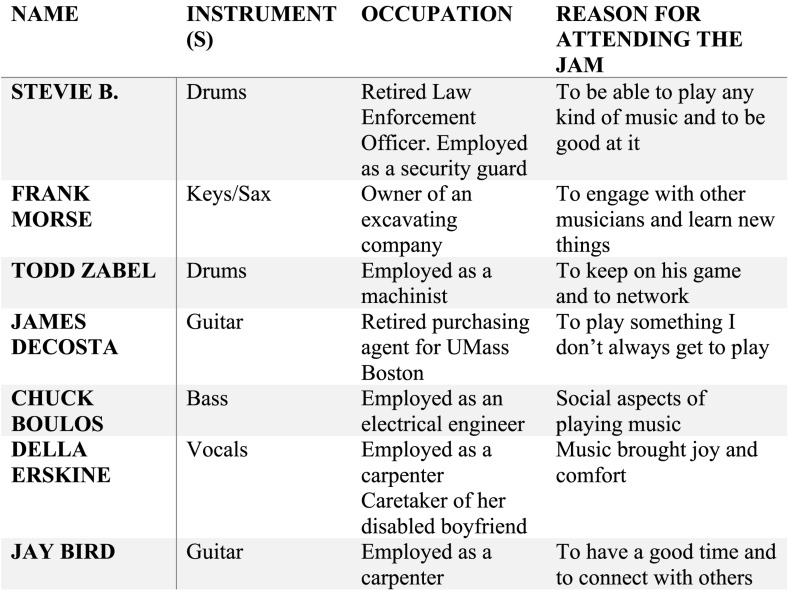
Characteristics of the participants in the study. The participants are listed in the order they were interviewed.

I began my data analysis by highlighting “first impression phrases derived from an open-ended process called initial coding” (Saldaña, 2009, p. 4). Next, I employed descriptive coding (Saldaña, 2009) to identify specific topics in the data. After completing this process, I was able to delineate a connection between the perspectives of the participants and a “larger theoretical framework” (Creswell, 2007, p. 162). Progressive focusing, based on the thoughts of the participants, led me to employ Ryff and Singer’s (2008) six core dimensions of eudaimonic well-being as a structure for my analysis. The results reflect my interpretation of the individualized perspectives of the seven older musicians who participated in the study (see Figure 2).

### The Participants

Electrified forms of pop and rock music figured prominently in the musical biographies of the participants. Stevie B, a retired law enforcement officer, told me that he started playing drums during elementary school and that his skill level improved considerably after he started taking private lessons. Stevie B’s teacher taught him to play blues-based rock songs by The Rascals, Led Zeppelin, and Cream. During junior high, Stevie started playing in bands. Stevie B. talked a lot about his early experiences; how he enjoyed being in a band, how it was a thrill to be on stage, how music made him popular with girls, and about his fondness for silly songs like “Secret Agent Man.” By age 19, frustrated by the breakup of his band, Stevie B. sold his drums and didn’t play again for 43 years.

Frank Morse, leader of the band *Morse Code* became a musician in the fifth grade when he auditioned and got accepted into the school band. During high school, Frank hung out with friends and listened to jazz, in particular Miles Davis. Frank playing took off when he startedtaking saxophone lessons with “Boots” Mazulli, whose teaching was focused on improvisation and aural skills. After high school, Frank got drafted and served in Vietnam. When he returned home, he started an R&B band called *Wadell and the Undone*. As a young man, Frank played out four nights per week while holding down a day job as a mechanic to support his family.

Frank told me:

*I always leaned toward R and B and soul music because it’s just in me. It’s not so dry as some of the classic rock stuff is. Soul’s got an undertone going to it all the time and if you can ride that and feel that, it’s nice. It’s very comfortable. It’s a nice place to be.*

Frank never stopped playing music. He joked, “Probably never will stop. Pat me on the head with a shovel.”

Todd Zabel met Frank Morse at the jam, which is how he got a gig with *Morse Code.* As a child, Todd “beat on things when he heard music” so his mother bought him a drum set for Christmas. Todd did not get involved with music in school. He said, “I felt like in school it was all marching band and uh, you know, jazz band stuff and I wanted to play rock n roll.” Todd had to quite taking drum lessons because he did poorly in school. Ultimately, Todd learned to play drums by playing along with LP records by Kiss, The Cars, and Cheap Trick. Todd told me he loved playing the blues, and that bands like Led Zeppelin led him to discover artists such as Willie Dixon and Curtis Mayfield. Todd revealed that the secret of playing the blues and jamming was to “keep things simple and play a groove.”

James DeCosta arrived at the jam early each week so he could set up his amp and effects pedals on stage. When I asked him to tell me about himself, James began with, “I’m self-taught. I’ve been playing since I was about 19. I’m 66 and retired. So basically, it’s like some people have bowling night, I have music. It’s just that simple, you know.” James’ interest in guitar playing began with the Beatles, which prompted his parent’s to purchase a Silvertone electric guitar from Sears. He learned about the blues by hanging out with friends. When I asked about his musical influences, James said:

*The British blues guys were my thing. Blues breakers, you know John Mayall, Clapton, all those guys. The Yardbirds, Jeff Beck, all those guys. Besides, it was more of an edgy kind of blues that I liked. BB King and Freddy King and Muddy Waters too.*

James was not in a band, but he attended as many as three different jam sessions each weekend.

Chuck Boulos was still working as an electrical engineer, but he was thinking about retirement. He spent weekends gigging with a heavy, classic-rock band called *Shameless*. As a child, Chuck wanted to play bluegrass, so his parents bought him a four-stringed banjo. Frustrated with the banjo, Chuck picked the bass when he heard metal bands like Deep Purple and Black Sabbath. During college days, Chuck played in bands and gigged in the Boston area. After college, he “just messed around in the basement, playing along with albums and CD’s.” About 15 years ago Chuck decided to “get serious,” and found a band by attending a local jam. For Chuck, music was primarily a social activity. He was looking toward retirement and thinking about relocating. His musical goals entailed developing a “new circle of musicians” to hang out and jam with.

Della Erskine grew up singing on a farm in rural Carver MA. She described her childhood as:

*“…all cranberry bogs, you know, scooping cranberries, raising chickens and pigs and all that stuff. I don’t know if it’s still standing, but our house had an outhouse. We didn’t have a traditional bathroom until high school, after we got an apartment in town. Didn’t graduate. I did not graduate from high school because of a reading disability.”*

Della attended school in Plymouth where she sang classical, jazz, and pop music in the choir. Outside of school, R&B, soul, and the music of Motown inspired her to become a performer. After leaving high school, Della moved to Somerville, got a waitress job, and started singing in nightclubs. She preferred doing jazz, something quiet and mellow, but she was also comfortable with the blues. Her friends said, “You know Della, you’d be a great blues singer,” so she learned blues standards and got gigs singing with local bands in the Boston area.

The youngest participant, Jay Bird (nickname), met Hot Rod Billie at another jam, which is how he found his way to The Tavern. Jay Bird considered himself to be a “second-generation blues musician.” Although he grew up listening to alternative rock in the ‘90’s, Jay Bird learned songs by John Lee Hooker, T Bone Walker, and British rock groups like the Kinks and Cream when he and his friends discovered a parent’s vinyl record collection. Now, at age 42, after raising a family, Jay Bird was looking to get back into music. Jay Bird was introspective about his relationship with music saying, “I find that fascinating that your brain is kind of wired to music and the relationship of music to what’s going on in your life is very connected.”

## Findings

I sought to know how biographical factors and engagement with music influenced the lives of older adult blues/rock musicians. In the following section, I use the words of the participants to illustrate how lived experiences in and through music fostered a sense of well-being.

### Individual Choice and Autonomy

The ability to make individual choices about “how to manage one’s life and surrounding world” is a key aspect of subjective well-being (Ryff and Keyes, 1995, p. 720). *Environmental mastery* suggests being able to self-select a particular lifestyle or seek surroundings that satisfy specific needs (Deci and Ryan, 2000; Strauser et al., 2008). Frank’s needs were centered on music-making and learning. He said, “I picked up a lot tonight. When you go to a jam, you’re playing with different quality musicians. Some very good, some not so good. It’s amazing how you can put this hodge-podge together. I mean and make music.” James needed to be challenged by the musical interaction. He explained, “And so I just enjoy this kind of thing. It’s like jump into the fire and do the best you can; try and fit in with the other people around you.”

Participation in the jam reflected an autonomous approach to music-making. Autonomy refers to the “sense that one’s actions are freely chosen and reflect what one wants to do” (Kuykendall et al., 2018, p. 4). During the jam, everyone freely interpreted songs as they wished, and played off of each other during musical performances. Stevie B. told me he could play drum covers like the record, but preferred to “do his own thing. I’ll keep it solid, but I do what I want to do.” Todd said he made the songs his own by “putting in what I feel.” James explained that he was all about “doing his own thing.” He said, “I’m like improvisation all the way. I’m making shit up all the time. I do everything I want to do, the way that I want it.” Della’s performing style was spontaneous and interactive. Her improvisatory style necessitated that the band pay close attention when she sang. She admitted that she had a tendency to “ad lib the lyrics a lot.” The impromptu atmosphere of the jam enabled each musician the opportunity to fulfill individual musical needs and to express divergent artistic ideas.

### Acceptance of Self and Others

Jamming engendered feelings of affirmation, accomplishment, and self-acceptance. Self-acceptance is a construct that involves having a realistic, albeit subjective, “awareness of one’s strengths and abilities” (Shepard, 1979, p. 139). Mastering performance goals and learning goals leads to self-acceptance and the acceptance of others (Deci and Ryan, 2000).

Stevie B. arrived at the Tavern early every Sunday so that he could get a table directly in front of the stage. He told me that in his retirement, playing the drums gave him a sense of accomplishment. The first time he got up on the stage to play at the jam, it ended badly. He hadn’t played for 43 years, so his lack of practice combined with alcohol consumption, caused him to play poorly. When Stevie got off the stage that night, members of the house band gave him looks of disapproval and offered to drive him home. It was discouraging. Stevie B. recognized that music provided him with the sort of challenges he needed in his life. His goal was to improve his playing so he could drive around, “play anywhere” and “prove to everybody how good I am.”

James DeCosta contended that his musical strength was his ability to improvise. Todd described his drumming as “solid” and that his power was “playing a groove.” Chuck Boulos expressed that playing bass was “one of the few things I do really well.” Della felt accomplished at connecting with her audience, saying, “I think making just people smile is the gift that I give.” Jay Bird was looking for his musical niche. Constructs associated with self-acceptance have been linked to well-being and in turn, living a “good life” (King and Napa, 1998; Twenge and King, 2005).

### The Benefits of Music Participation

The relational benefits of music participation have been documented in the literature (Laukka, 2007; Gembris, 2012). Music participation at the jam fostered personal connections. Stevie B. knew just about everyone by name. Chuck said, “there’s good friendship here. It’s not clique-y like some jams.” Todd said he came to the jam to “check it out. All I was going to do was check it out. I wasn’t in the room 10 min and my name was on the list. I’ve been coming here ever since.” Chris, the bass player in the house band, called James up and said: “Hey I’m runnin’ a jam down in Norton. You wanna go? That was 6 or 7 years ago and I’ve never stopped coming.” Jay Bird said he came back to The Tavern because “he got the vibe that this place was somewhere that people could come and have a good time.”

Viewing one’s life as having meaning, purpose, direction, and goals is perceived as the existential core of eudaimonic well-being (Ryff, 2018). Stevie B. was very specific regarding the purpose of music in his life. When Stevie retired, he needed an activity that would get him off the couch and away from alcohol, television, and the internet. His wife gave him a year to straighten himself out, so he started taking drum lessons at a local music store. He told me, “It’s [music] helped me stay focused and helped me relate [to other people]. No matter what I do, how good I get, I have to go even further, to be as good, if not better than, John Bonham (Led Zeppelin), Buddy Rich, and Neil Peart (Rush). That’s my goal.”

Frank stated that his reasons for attending the jam were related to his need for musical growth. He said, “A musician will always continue learning. If not, then he’s kidding himself or herself, okay?” Chuck’s purpose for attending the jam had to do with long-term musical goals and objectives. He said, “After I get into my sixties, I still want to play. I actually have a goal when I retire, to play drums.” James exclaimed, “I just love to play! It’s like being addicted to drugs, but much more healthy [sic]. I get to go out and do my thing. Keep my chops up and practice my craft.” Jay Bird revealed he was at the jam “hoping to find something that kind of takes a little left turn to something that is my own.”

Older adults tend to experience feelings of tranquility and transcendence through music participation more often than younger people (Ellermann and Reed, 2001; Minichiello and Hayes, 2005). Each participants expressed the ways music gave them a personal sense of joy, belonging, and feelings of transcendence. Della told me, “I look for the joy in it. It’s all about the therapeutic stuff when it comes to music.” Chuck said, “I just love to play. I just … I just go in another world.” Jay Bird offered, “music can earmark certain points in your life. Like you hear a song and you’re transported back to that point in time. Todd said, “Music helps me through my day and my life. Music is what keeps me going.” Frank believed music was a gift. He said, “God’s been good to me. He gave me a lot of talent.” The personal growth that happens in later life can be affirming as well as transformative (Bauer et al., 2015).

## Limitations

This study reflects my own desire to understand the eudaimonic benefits of music participation among older adults at a local jam. Certain limitations influenced the results. I acknowledge that the sample size was small, participant selection was a matter of convenience, and the study took place over a relatively brief period of time at a single location in the Northeast region of the United States. Furthermore, this report is based on my interpretation of the data, and is a reflection of “how the research experience affected me” (Wolcott, 1994, p, 44). Despite these constraints, this study represents an initial step in an effort to understand how music participation may help people lead better, more satisfying lives.

## Discussion

The outcomes of this investigation suggest that participatory forms of music-making integrate values of autonomy and interconnectedness, freedom of expression combined with responsibility to others, sonic and spatial awareness, creativity, and tradition. Although they explained it in different ways, the participants indicated that music participation contributed to their social, emotional, spiritual and psychological well-being. Music was important, not just as a form of entertainment, but as an integral part of the fabric of each of the participants’ lives, defining their social and personal identities (Hargreaves et al., 2002). The participants communicated who they were, and how they wished to be perceived by others, in and through participatory music-making.

The goal of jamming is to entice people to join in for the purpose making one’s self and other people feel good. Turino (2008) contended that “Art is not really an “imitation of life”; it would be more accurate to say that artistic processes crystalize the very essence of a “good life” (Turino, 2008, p. 18). Such artistic experiences combine an interplay of ideas that draw from what we already know to create imaginative possibilities in the moment. Successful artistic experiences and performances are based on artistic interplay and often, for a brief moment, we feel a sense of elation and timelessness. The innate need (Deci and Ryan, 2000) for a sense of accomplishment, acceptance, and belonging motivates humans to engage in forms of participatory music-making which, in turn, engenders well-being.

### Educational Implications

A jam is an open-ended, informal participatory musical event predicated on aural, improvisatory, informal, small-group engagement. The music is selected by the participants. There is no rehearsal, nor are there predetermined musical arrangements or prescribed learning outcomes. The outcomes of jamming are predicated on in-the-moment collective musical interpretation and spontaneous collaboration. From an educational perspective, engagement with music through socially shared experiences, results in the individual acquisition of learning strategies and new musical knowledge.

Each participant took an individualized, self-directed approach to music learning. Self-Directed Learning (SDL) is defined as any “increase in knowledge, skill, accomplishment, or personal development that an individual selects and brings about because of his or her own efforts” (Gibbons, 2002, p. 2) Rooted in cognitive and developmental psychology, SDL is centered on the social and emotional needs, learning interests, and abilities of the individual. A basic assumption of SDL is that people live lives of learning and learn to live well. SDL is focused on “personal growth, transformations in perspective, and self-actualization” (Creech et al., 2020, p. 205). Self-actualized people function autonomously, are able to set personal goals, and assume responsibility for their own emotional and physical well-being.

Artistic processes that lead to a “good life,” are embodied during music-making.

Like Della said, “There’s that one song you will hear, and it will just trigger a good, joyful feeling inside of you.” Music classrooms, community music settings, and private studios have the potential to be sites of musical and intersubjective interaction where people can experience “happiness, flow, fellowship, care for others, well-being, and many other attributes of eudaimonia” (Elliott and Silverman, 2014, p. 70). Drawing from the philosophical ideas of contended that both “musical artistry *and* eudaimonia are among the ultimate aims of music making and music teaching and learning” (p. 59).

## Conclusion

This study emerged from my desire to explore ways that well-being, in and through music, is central to living a “good life.” Well-being is “at the heart of artistry and what it means to live rightly in the world with others” (Smith and Silverman, 2020, p. 1). It is important to remember that a “good life is a process, not a state of being. It is a direction, not a destination” (Rogers, 1976, p. 187). Frith (1996) suggested, “art is not a way of expressing ideas; it is a way of living them” (p. 111). The findings of this study suggest that eudaimonic well-being is the result of active engagement in human activities that are goal-directed and purposeful, and a good life involves the self-realization of individual dispositions and talents over a lifetime.

Understanding the variety of ways that older people perceive themselves in relation to music has the potential to explain how engagement with music and music learning may to contribute to a better quality of life during the lifespan of individuals of all ages. Perhaps this investigation will inspire future research studies on eudaimonic well-being within a broader range of popular and community-based music settings that include people from a variety of age groups and geographic locations. It is my hope that this study will inspire individualized, improvisatory approaches in music classrooms as well as other contexts where humans embody participatory music-making as an act of joy.

## Data Availability Statement

The raw data supporting the conclusions of this article will be made available by the author, without undue reservation.

## Ethics Statement

The studies involving human participants were reviewed and approved by the Boston University IRB Protocol #5312X. The patients/participants provided their written informed consent to participate in this study. Written informed consent was obtained from the individual(s) for the publication of any potentially identifiable images or data included in this article.

## Author Contributions

The author confirms being the sole contributor of this work and has approved it for publication.

## Conflict of Interest

The author declares that the research was conducted in the absence of any commercial or financial relationships that could be construed as a potential conflict of interest.
